# Selective Dye Adsorption and Antimicrobial Performance of Cellulose–Chitosan Hydrogels and Aerogels: Role of Supramolecular Organization

**DOI:** 10.3390/polym18131649

**Published:** 2026-07-02

**Authors:** Cristóbal Donoso, Isidora Reyes-González, Katherine Sossa Fernández, Javier Coronil, Pablo Reyes-Contreras, Isabel Carrillo-Varela, Benjamín Opazo, Rodrigo Hasbún, Regis Teixeira Mendonҫa

**Affiliations:** 1 Facultad de Ciencias Forestales, Universidad de Concepción, Concepción 4030000, Chile; cdonoso2020@udec.cl (C.D.); ireyes2017@udec.cl (I.R.-G.); ksossa@udec.cl (K.S.F.); 2 Laboratorio de Biopolímeros y Materiales Biobasados, Centro de Biotecnología, Universidad de Concepción, Concepción 4030000, Chile; preyesc@udec.cl; 3 Laboratorio de Biopelículas y Microbiología Ambiental, Centro de Biotecnología, Universidad de Concepción, Concepción 4030000, Chile; jcoronilcarrillo@yahoo.com; 4 Centro de Innovación en Tecnología y Diseño de Materiales para el Entorno Construido—CIMAC-ANID CTI250005, Santiago 9170022, Chile; 5 Bioforest SpA, Camino A Coronel Km 15, Coronel 4090000, Chile; isabel.carrillo@arauco.com; 6 Programa de Doctorado en Ingeniería, Facultad de Ingeniería, Universidad del Bío-Bío, Concepción 4030000, Chile; benjamin.opazo2001@alumnos.ubiobio.cl; 7 Laboratorio de Epigenética Vegetal, Departamento de Silvicultura, Facultad de Ciencias Forestales, Universidad de Concepción, Concepción 4030000, Chile; rodrigohasbun@udec.cl

**Keywords:** cellulose, chitosan, hydrogels, aerogels, dye adsorption, ionic liquid

## Abstract

Cellulose and chitosan are biopolymers widely used to prepare composites due to their complementary charges and intrinsic biocompatibility. While they are mainly of interest for medical applications, they are also suitable for water remediation. In their native states both biopolymers are non-porous; however, after dissolution and subsequent regeneration they can form porous structures that are better suited for such applications. In this work, cellulose pulp and chitosan were dissolved in an ionic liquid and regenerated in water at different mass ratios to produce hydrogels and their corresponding aerogels. The materials were structurally characterized and evaluated for dye adsorption and antimicrobial performance. Methylene blue and Congo red were selected as cationic and anionic dyes, respectively. The concentrations went from 5 to 80 mg/L in 24 h batch adsorption experiments. Chitosan-rich and intermediate cellulose–chitosan hydrogels preferentially removed Congo red, reaching 27 ± 1 mg/g and 24 ± 1 mg/g at 80 mg/L, respectively; the fully cellulose hydrogel maximized methylene blue uptake, achieving 23 ± 1 mg/g under the same conditions. SEM and XRD analyses revealed a hybrid architecture in which chitosan coats cellulose fibers and becomes more amorphous, while cellulose preserves crystalline domains that act as a rigid, highly porous backbone. Aerogels derived from freeze-dried hydrogels exhibited high porosity and water uptake, together with broad-spectrum antimicrobial activity, achieving bactericidal levels (≥99.9% inhibition) against *Staphylococcus aureus* for all compositions and against *Escherichia coli* for selected cellulose–chitosan ratios. These results demonstrate that cellulose–chitosan hydrogels and aerogels function as multifunctional bio-based materials whose supramolecular organization, surface charge distribution, and porosity can be tuned to balance adsorption selectivity and antimicrobial performance for advanced environmental applications.

## 1. Introduction

Natural polymers, such as polysaccharides, are common components on Earth, forming a wide range of substances with chemical structures formed by the association of numerous identical small molecules through glycosidic bonds [1]. This structure allows high biocompatibility, biodegradability, non-toxicity and modification capacity, making them ideal for the fabrication of a wide variety of industrial and biomedical products [2,3]. The most abundant polysaccharide on Earth is cellulose, which is composed of a linear structure of glucose units connected by β-glycosidic linkages. The three hydroxyl groups at C2, C3 and C6 of each glucose unit allow the formation of strong intermolecular and intramolecular hydrogen bonds, forming cellulose layers that give rise to microfibrils and fibers [4,5]. This network of bonds not only defines the layered structure of cellulose but also gives it excellent mechanical strength [6]. On the other hand, chitosan is a natural polysaccharide derived from chitin, which is found in crustacean shells, insect cuticles and fungal cell walls. It is mainly composed of glucosamine units obtained by N-deacetylation of chitin through an alkaline treatment [7]. The glucosamine units have hydroxyl groups at C6 and C3, primary and secondary respectively, highlighting the characteristic primary amine group at C2. This antimicrobial activity of chitosan is mainly due to the interaction of its protonated amino groups with the cell membranes of microorganisms [8,9]. In terms of chemical nature, chitosan and cellulose have a similar structure, differing only in the functional group on the C2 carbon, where in chitosan it is a primary amino group that makes the particle positively charged, instead of the hydroxyl groups of cellulose, which render the polymer negatively charged [10]. The mixing process between these polymers is known to cause a disruptive effect on crystallinity, resulting in disruption of the original crystalline organization and the formation of an intermixed polymer network [11].

The combination of cellulose and chitosan can serve as a material platform for a wide range of derivatives, where both polysaccharides’ properties are needed. Polymer hydrogels are widely used in agriculture, the food industry, drug delivery and even medicine [12]. Increasing interest in polysaccharide-based hydrogel production has been driven by environmental concerns [2,13]. This material is a three-dimensional network composed of hydrophilic polymer chains held together by physicochemical cross-links, which allow them to absorb and retain a significant amount of water in their structure without dissolving [14]. Either chitosan or cellulose can be used as the main component of a hydrogel. However, it is usually mixed with other components such as acrylamide, PEG and PLA to improve the dye adsorption capacity and the thermo-mechanical stability of the material [15,16,17]. When chitosan and cellulose are subjected to a dissolution–regeneration cycle, they create a polymeric network, made by extensive hydrogen bonding and physical entanglement, without the need for covalent cross-linkers. This type of interaction can result in hydrogels with high porosity and efficiency in the removal of dyes [18].

In recent years, numerous cellulose–chitosan hydrogels and aerogels have been developed as bio-based adsorbents and functional porous materials. Several studies have reported composite aerogels based on cellulose nanofibers or nanocrystals and chitosan for the removal of anionic dyes and heavy metal ions from water, as well as for oil/water separation and water disinfection, highlighting the role of cellulose as a rigid, highly porous scaffold and chitosan as a source of functional amino groups for contaminant binding [19,20,21,22]. Other works have exploited cellulose–chitosan aerogels as hybrid sorbents for volatile pollutants such as formaldehyde or as antibacterial supports, showing that the combination of both polysaccharides can simultaneously provide large surface area and bioactivity [23,24]. However, most of these studies focus either on maximizing adsorption capacity by introducing additional components or on a single target pollutant, and only partially address how the cellulose–chitosan ratio and the resulting supramolecular organization control porosity, crystallinity and charge distribution in a systematic way.

In this work, cellulose pulp and chitosan are co-dissolved and regenerated in an ionic liquid at five different ratios to produce a group of hydrogels and aerogels in which the composition is the only varying parameter. By coupling density and porosity measurements with XRD, optical microscopy and SEM, and by evaluating the adsorption of a cationic dye (methylene blue) and an anionic dye (Congo red) under controlled conditions, this study aims to elucidate how the cellulose–chitosan ratio and the associated hybrid architecture govern dye selectivity and water uptake. This approach is based on the use of a two-component system processed via ionic liquid dissolution and regeneration to decouple structural effects from additional chemical modifications, thereby providing a structure–property framework for designing cellulose–chitosan hydrogels and aerogels for environmental remediation applications.

## 2. Materials and Methods

### 2.1. Materials

**Figure 1:**
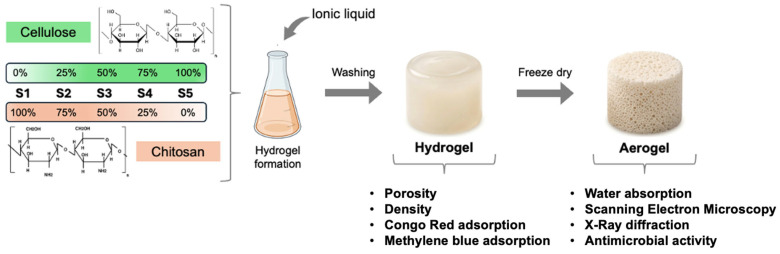
Methodological scheme for the preparation and characterization of cellulose–chitosan hydrogels and aerogels.

Bleached eucalyptus kraft pulp (80% Eucalyptus nitens and 20% Eucalyptus globulus) was supplied by a local Chilean pulp mill. Low-molecular-weight chitosan (50–190 kDa) was purchased from Sigma-Aldrich (St. Louis, MO, USA). The ionic liquid 1-ethyl-3-methylimidazolium chloride (EMIMCl) was obtained from Sigma-Aldrich (St. Louis, MO, USA). Methylene blue (≥95%, Merck, Darmstadt, Germany) and analytical-grade Congo red (Sigma-Aldrich, St. Louis, MO, USA) were used as model cationic and anionic dyes, respectively. Distilled water was used in all preparation, washing and adsorption steps. Tryptic soy broth (TSB), tryptic soy agar (TSA) and PBS-Tween for microbiological assays were commercial dehydrated media purchased from Winkler (LINSAN brand, Santiago, Chile) and from Sigma-Aldrich (GranuCult brand, St. Louis, MO, USA), and prepared according to the manufacturers’ instructions. General scheme for preparation and characterization of hydrogels and aerogels is shown in Figure 1.

### 2.2. Preparation of Cellulose–Chitosan Hydrogels

Five types of cellulose–chitosan hydrogels with different polymer ratios (Table 1) were prepared in triplicate by dissolution and regeneration in 1-ethyl-3-methylimidazolium chloride. Dissolution was performed in 20 mL glass beakers containing 10 mL of ionic liquid, placed on a magnetic hot plate stirrer set to approximately 110 °C and 300 rpm. Once the ionic liquid reached the target temperature, the required amount of chitosan for each formulation was slowly added while stirring until no undissolved particles were visible. Then, the corresponding amount of cellulose pulp was introduced in the same way, and stirring was continued until a uniform, viscous yellowish and transparent gel was obtained. The solutions were allowed to cool to room temperature to form solidified gels, which were subsequently regenerated by volumetric dilution with distilled water. The hydrogels were transferred to an orbital shaker and washed with repeated water changes over several days. Washing was continued until the supernatant became odorless and its conductivity reached values comparable to distilled water and no further decrease was observed.

### 2.3. Hydrogel Characterization

#### 2.3.1. Density

The hydrogel density (p) was calculated using Equation (1).

(1) p=mV

where *m* and *V* correspond to mass (g) and volume (cm^3^) of the hydrogel [25].

#### 2.3.2. Porosity

The porosity (P) of each sample was calculated according to Equation (2).

(2) P%=(Ww−Wdry)ρH2O(Ww−Wdry)ρH2O+Wdry/(wc/ρc+wch/ρch)×100

where *Ww* and *Wdry* correspond to the moisture and lyophilized mass (g) of the hydrogel respectively, *ρH_2_O* is the density of water (0.998 g/cm^3^), *wc* and *wch* are the mass fractions of cellulose and chitosan in the hydrogel, *ρc* is the density of cellulose (1.528 g/cm^3^) and *ρch* is the density of chitosan (1.35 g/cm^3^) [26,27].

#### 2.3.3. Methylene Blue and Congo Red Adsorption

Adsorption tests were carried out in duplicate in Falcon tubes containing 5 mL of dye solution at initial concentrations of 5, 10, 25, 50 and 80 mg/L. A dry mass of 0.03 g of never-lyophilized hydrogel was added to each tube, and the suspensions were agitated on an orbital shaker at 200 rpm for 24 h at room temperature. The residual dye concentration was determined using a UV–Vis spectrophotometer (UV-1280, Shimadzu Corporation, Kyoto, Japan) at 664 nm for methylene blue and 500 nm for Congo red (corresponding to their maximum absorbance peaks). The equilibrium adsorption capacity *qe* (mg/g) was calculated using Equation (3).

(3) $$qe=\frac{\left(C0-Ce\right)V}{m}$$

where *C*0 and *Ce* are the initial concentration and equilibrium concentration of MB, respectively (mg/L), *V* is the volume of the solution (L) and *m* is the dry mass of the hydrogel (g) [5].

#### 2.3.4. Aerogel Preparation

To obtain the aerogels, the hydrogels were frozen at −18 °C and then dried in a freeze dryer (Martin Christ Gefriertrocknungsanlagen GmbH, Osterode am Harz, Germany) at a condensator temperature of −85 °C with a vacuum setting of 0.055 mbar for 24 h. The aerogels were stored in a dry place at room temperature until characterization.

### 2.4. Aerogel Characterization

#### 2.4.1. Water Absorption Capacity

The samples were exposed to distilled water for 14 days and the tests were performed in triplicate. The water absorption (S) was obtained from Equation (4).

(4) $$S\left(\%\right)=\frac{\left(Wsw-Wdry\right)}{Wdry}\times 100$$

where *Wsw* is the wet mass (g) after removal of surface distilled water and *Wdry* is the lyophilized mass (g) before absorption [25,28]. After the initial measurements, the samples were lyophilized and then subjected to rehydration in distilled water for 30 days at room temperature.

#### 2.4.2. Scanning Electron Microscopy

Aerogel samples were mounted on a specimen stub and were coated with gold for 60 s using a metallizer sputter coater (SPI-MODULE, SPI Supplies, West Chester, PA, USA). Images were obtained using a scanning electron microscope (JSM-6380LV, JEOL Ltd., Tokyo, Japan) connected to a computer for image acquisition. SEM micrographs were taken at magnifications of 30×.

#### 2.4.3. X-Ray Diffraction (XRD)

The samples were taken to the hydraulic press to obtain pellets for XRD analysis [29]. Measurements were performed at an angle of 2θ, with an estimated range of 3° to 60°, to assess the crystalline/amorphous character of the polymers, and the diffractogram patterns were fitted using the PeakFit V4.12 PeakFit software, version 4.12 (Systat Software Inc., San Jose, CA, USA). By applying deconvolution to the XRD peaks, the crystallinity index of the polymers was calculated using Equation (5):

(5) $$CrI\left(\%\right)=\;\frac{Acryst}{Atotal}\times 100$$

where *A_Cryst_* is the sum of the crystalline areas and *A_total_* is the total area provided by the diffractogram [5,30]. The crystallite size (*L*) was then calculated using Equation (6):

(6) $$L=\frac{k\times \lambda }{\beta \times \cos\theta }$$

where *L* is the crystallite size (nm), *k* is Scherrer’s constant (0.96), *λ* is the wavelength, *β* is the full width at half maximum (FWHM) and *θ* is the Bragg angle, corresponding to the (200) and (110) planes for cellulose and chitosan, respectively [31].

#### 2.4.4. Antimicrobial and Biofilm Assays

The antimicrobial performance of the cellulose–chitosan aerogels was evaluated against a Gram-negative strain (*Escherichia coli*) and a Gram-positive strain (*Staphylococcus aureus*), using a time–kill assay adapted from [32,33] with modifications. Briefly, each strain was pre-cultured in tryptic soy broth (TSB) at 37 °C for 24 h under constant agitation and adjusted to a turbidity equivalent to 0.5 McFarland (≈1.5 × 10^8^ CFU/mL). The standardized suspensions were prepared such that the addition of 100 µL of the suspension to 5 mL of sterile TSB in 15 mL Falcon tubes containing one aerogel specimen resulted in a final concentration of approximately 3 × 10^5^ CFU/mL; tubes without aerogels served as bacterial controls.

Prior to inoculation, aerogels were disinfected inside a laminar flow cabinet by spraying with 70% (*v*/*v*) ethanol, allowing for complete evaporation, and exposing both sides to UV light for 30 min. After inoculation, tubes were incubated at 37 °C, and aliquots were taken at 24 h, followed by serial ten-fold dilutions in sterile distilled water. From each appropriate dilution, 20 µL were plated on tryptic soy agar (TSA) by the microdrop method and incubated at 37 °C for 24 h, and viable cell counts were determined from plates containing 30–300 colonies, expressed as CFU/mL.

For biofilm cell counting, at 24 h, the liquid medium was completely removed to assess biofilm formation on the aerogel surface. The aerogels were transferred to tubes containing 5 mL of PBS-Tween and sonicated for 5 min in an ultrasonic bath to detach adherent cells, followed by serial dilutions and microdrop plating on TSA as described above to quantify biofilm-associated CFU/mL. As a biofilm control, glass was used as an inert, non-porous support under the same conditions. All counts were performed in triplicate.

The inhibitory rate (%) for planktonic cells at 24 h was calculated relative to the corresponding bacterial control according to Equation (7):

(7) $$Inhibitory\;rate\left(\%\right)=\left(1-\frac{N_{t}}{N_{0}}\right)\times 100$$

where $N_{t}$ is the viable cell count (CFU/mL) in the presence of the aerogel and $N_{0}$ is the viable cell count (CFU/mL) in the control without aerogel at 24 h. In this work, reductions ≥ 99.9% in planktonic cells (corresponding to ≥3 log units) were considered indicative of a truly bactericidal effect, whereas lower reductions were interpreted as growth inhibition without complete killing, consistently with criteria used for nanoparticle-based antimicrobial systems.

## 3. Results and Discussion

### 3.1. Hydrogel Characterization

**Figure 2:**
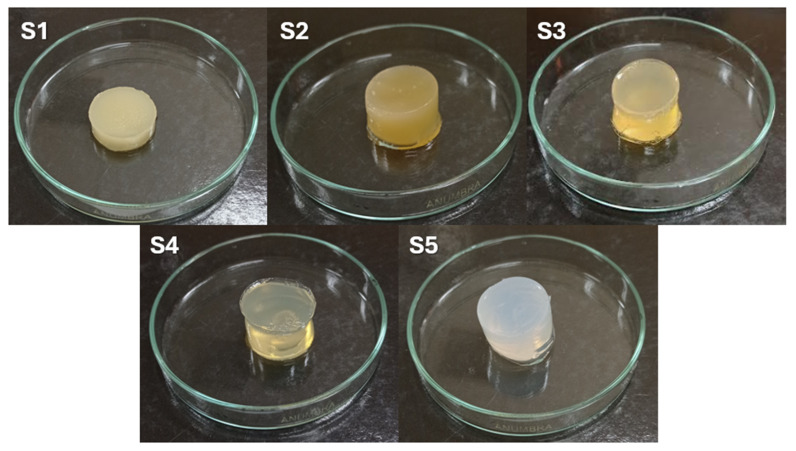
Cellulose–chitosan hydrogels prepared at different cellulose/chitosan mass ratios, S1 (0/100), S2 (25/75), S3 (50/50), S4 (75/25) and S5 (100/0), as summarized in Table 1.

The hydrogels produced using different proportions of cellulose and chitosan (Table 1) are shown in Figure 2, while their porosity and density are presented in Table 2. Sample S1, composed of 100% chitosan, exhibited the highest density and one of the lowest porosities. As cellulose was incorporated, the density of the hydrogels decreased and remained similar among the cellulose-containing samples, whereas the porosity showed the opposite trend, increasing up to a maximum in sample S3, which contained 50% cellulose and 50% chitosan. This behavior is consistent with previous reports for cellulose–chitosan aerogels with a 1:1 ratio, as described by Duong et al. [34]. In the present system, this trend must also be considered in the light of different chain architectures of the two polysaccharides. The chitosan used here is a low-molecular-weight-grade, whereas the regenerated cellulose pulp consists of longer, more crystalline chains that act as a rigid backbone. Carrillo-Varela et al. reported that during dissolution and coagulation in the ionic liquid, the effective cross-linking density therefore depends not only on composition but also on polymer chain length [27]. Consequently, the inverse correlation between density and porosity observed for our mixtures reflects a balance between compact domains associated with low-molecular-weight chitosan and the more extended network supported by longer cellulose chains, in agreement with previous reports on cellulose–chitosan hydrogels and aerogels [35].

**Figure 3:**
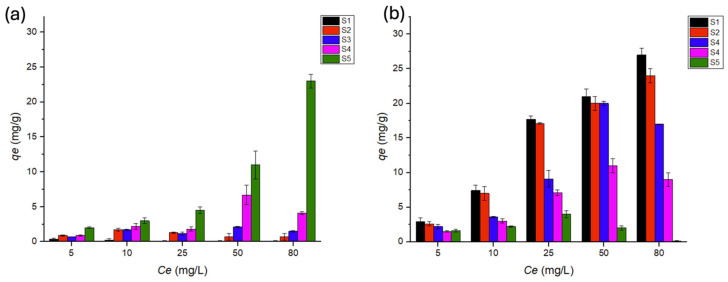
Adsorption capacity *qe* of cellulose–chitosan hydrogels with different cellulose/chitosan mass ratios: S1 (0/100), S2 (25/75), S3 (50/50), S4 (75/25) and S5 (100/0). (**a**) Adsorption capacity *qe* of methylene blue. (**b**) Adsorpcion capacity *qe* of Congo Red.

The dye adsorption capacity of the cellulose–chitosan hydrogels was evaluated using batch experiments with Congo red (anionic dye) and methylene blue (cationic dye) at equilibrium after 24 h of exposure to different dye concentrations (5, 10, 25, 50 and 80 mg/L). In this concentration-dependent assay, chitosan-containing hydrogels exhibited a clear preference for Congo red, whereas cellulose-rich compositions preferentially adsorbed methylene blue (Figure 3).

For Congo red, samples S1, S2 and S3 showed the highest adsorption capacities over the entire concentration range. At low concentrations (5 and 10 mg/L), Congo red uptake already reached 2.9 ± 0.6 to 7.4 ± 0.8 mg/g in S1 and remained above 2.5 mg/g for S2 and S3, while at 25 and 50 mg/L the adsorption increased markedly, attaining values close to 17–21 mg/g. At the highest concentration (80 mg/L), S1 and S2 reached 27 ± 1 and 24 ± 1 mg/g, respectively, and S3 remained close to 19 mg/g, confirming that the presence of chitosan promotes strong interaction with the anionic dye throughout the evaluated range. Due to its anionic nature, Congo red is attracted by the cationic charge of the primary amine group of chitosan, which explains the higher adsorption observed in chitosan-rich and intermediate cellulose–chitosan hydrogels [36].

In contrast, S4 and S5, which contained higher proportions of cellulose, displayed systematically lower Congo red adsorption capacities. S4 showed values between 1.5 ± 0.3 and 11 ± 1 mg/g depending on the concentration, whereas S5 decreased from 1.6 ± 0.2 mg/g at 5 mg/L to only 0.08 ± 0.1 mg/g at 80 mg/L. These results indicate that cellulose alone contributes little to the removal of this anionic dye under the tested conditions and that the availability of protonated amino groups in the hydrogel network is the main factor controlling Congo red uptake.

The adsorption of methylene blue showed an opposite tendency. The fully cellulose hydrogel (S5) exhibited the highest capacities at intermediate and high dye concentrations, reaching 4.5 ± 0.5 mg/g at 25 mg/L, 11 ± 2 mg/g at 50 mg/L, and 23 ± 1 mg/g at 80 mg/L, whereas S1—S4 remained at much lower levels, generally below 3 mg/g across the same concentration range. The increase observed for S5 with rising methylene blue concentration is consistent with the interaction between the hydroxyl groups present in cellulose and the positively charged methylene blue molecules, which provides favorable binding sites in the absence of competing cationic species [37].

For chitosan-containing hydrogels (S1–S4), methylene blue adsorption remained limited, with values typically between 0.08 and 2.9 mg/g even at the highest concentration evaluated. This marked reduction compared with S5 suggests that the incorporation of chitosan alters the surface charge distribution of the cellulose network. The positive amino groups of chitosan can interact with the negatively charged hydroxyl groups of cellulose, reducing their availability for binding methylene blue and giving rise to cellulose–chitosan complexes that are less efficient towards cationic dye uptake. In addition, the positive charge of chitosan tends to coat the cellulose molecules, masking their charge and further hindering methylene blue adsorption, even when chitosan is present at only 25% of the polymer fraction as that in S4 [38].

### 3.2. Aerogel Characterization

**Figure 4:**
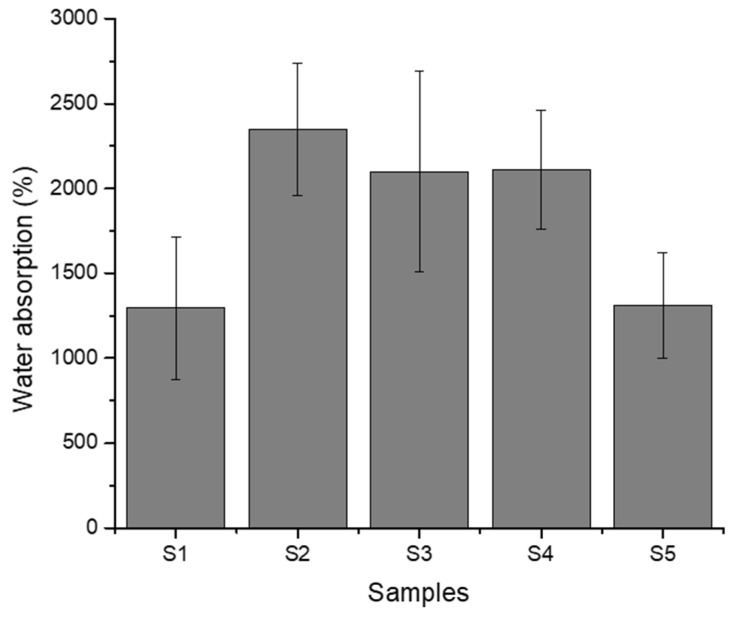
Water absorption of cellulose–chitosan aerogels obtained from hydrogels with different cellulose/chitosan mass ratios: S1 (0/100), S2 (25/75), S3 (50/50), S4 (75/25) and S5 (100/0).

The aerogels produced by the lyophilization of cellulose–chitosan hydrogels were characterized. The water absorption capacity of the aerogels was tested (Figure 4). Aerogels S1 and S5 presented low absorption compared to the hydrogels composed of both polymers, which is consistent with density and porosity values measured before lyophilization in the corresponding hydrogels.

**Figure 5:**
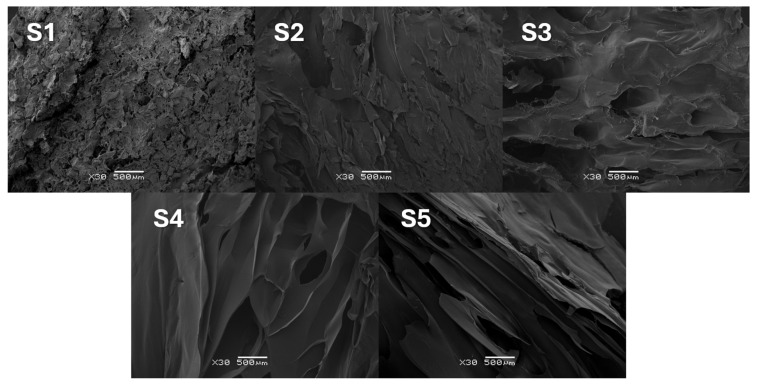
SEM images (30× magnification) of cellulose–chitosan aerogels with cellulose/chitosan mass ratios: S1 (0/100), S2 (25/75), S3 (50/50), S4 (75/25) and S5 (100/0).

The SEM images shown in Figure 5 illustrate how the cellulose–chitosan ratio affects the internal architecture of the aerogels. In S1 (0/100), the low-molecular-weight chitosan forms a compact, highly aggregated surface, consistent with the relatively high density and lower porosity of this composition. As the cellulose content increases (S2–S4), the internal structure evolves into a continuous, lamellar skeleton in which chitosan domains tend to coat and bridge the regenerated cellulose network, giving rise to elongated pore walls and channels that reflect the higher porosity and water absorption of the mixed systems. In S5 (100/0), the aerogel derived from pure cellulose exhibits a more regular and well-organized lamellar network. At the magnification used here (30×), the pore walls generated during freeze-drying coalesce into a coherent, interconnected framework, so that the aerogels are observed as continuous networks with a high internal void fraction rather than as loose aggregates of discrete particles, in agreement with the density, porosity (Table 2) and water uptake behavior (Figure 4).

**Figure 6:**
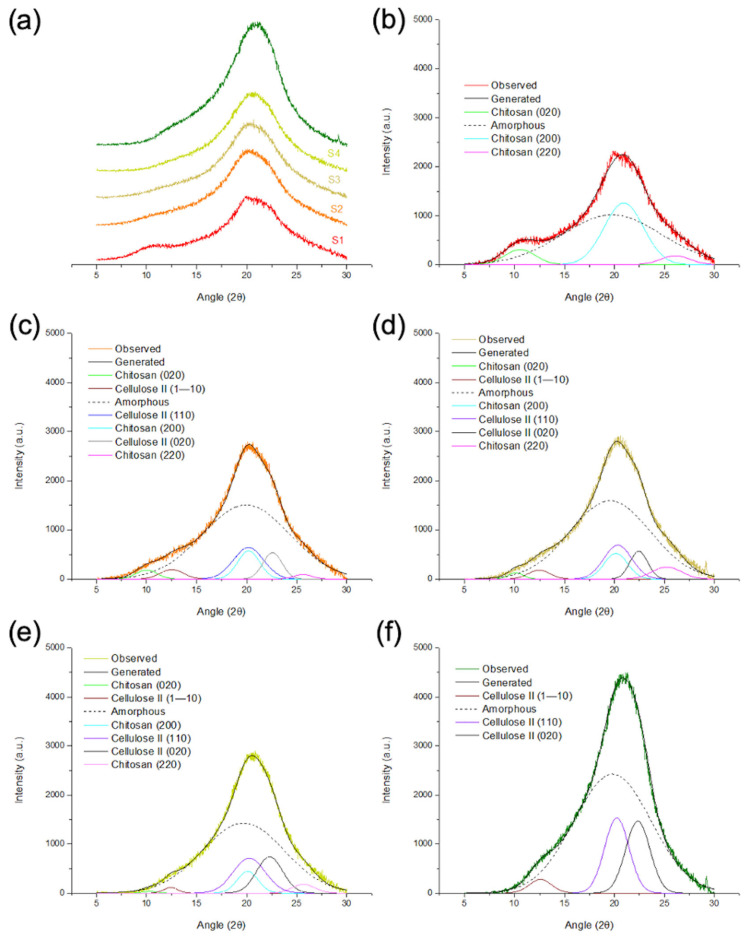
Observed and fitted XRD diffractograms of cellulose–chitosan aerogels with different cellulose/chitosan mass ratios. (**a**) Observed diffractograms of all aerogels S1–S5. (**b**) Deconvolution of the S1 (0/100) pattern into the observed profile and its fitted components. (**c**) Deconvolution of the S2 (25/75) pattern. (**d**) Deconvolution of the S3 (50/50) pattern. (**e**) Deconvolution of the S4 (75/25) pattern. (**f**) Deconvolution of the S5 (100/0) pattern showing the observed profile and its fitted components. Colored lines in panels (**b**–**f**) correspond to the individual crystalline peaks of cellulose II and chitosan, and the dashed line represents the amorphous contribution.

Sample S1 exhibited diffraction peaks at 10.3°, 20.1° and 25.8°, corresponding to the (020), (200) and (220) planes of chitosan, respectively, together with a broader contribution in the 15–16° region associated with its amorphous fraction [39,40]. In contrast, regeneration of cellulose in S5 promoted chain reorganization into the cellulose II structure, with characteristic reflections at 12.0°, 20.0° and 21.6° assigned to the (110), (100) and (020) planes, respectively, and a broader amorphous contribution centered at 18.5° [5]. The intermediate samples S2, S3 and S4 displayed diffraction patterns containing contributions from both polysaccharides, indicating the coexistence of cellulose-rich crystalline domains and more disordered chitosan regions within the hybrid network (Figure 6).

The crystallinity index and the crystallite lateral sizes are shown in Table 3. The aerogel based on 100% chitosan (S1) exhibited a crystallinity of 37%, which reduced to 12% for sample S2 made of 75% chitosan. When the chitosan interacts with cellulose fibers, it becomes more amorphous due to the disruption of hydrogen bonds, allowing it to wrap the more crystalline structure of cellulose (18.5%). This disruption is mainly caused by the electrostatic interactions between positively charged chitosan and negatively charged cellulose [41]. Conversely, the crystallite size of chitosan increases as its concentration in the matrix decreases. Due to structural adaptation, chitosan in smaller amounts tends to aggregate around cellulose, promoting a co-crystallization phenomenon [10]. Then, the crystallinity index of both chitosan and cellulose remained nearly constant between samples S2 and S3, indicating a structural equilibrium in this composition range. However, in sample S4, a slight divergence was observed: cellulose showed an increase in crystallinity, while chitosan exhibited a small decrease, suggesting a reorganization of the polymeric network that favors the ordering of cellulose chains over those of chitosan. Furthermore, as the amount of cellulose increased in sample S4, its crystallinity increased to 24.8%. This demonstrates that cellulose can continue to form new crystalline regions in the presence of chitosan. The sample consisting of 100% cellulose (S5) exhibited the highest percentage of crystalline cellulose (29.3 ± 0.9).

The crystallite size was determined from six characteristic diffraction peaks. In both polysaccharides, dissolution and regeneration in the ionic liquid reduced the crystallite dimensions, reflecting partial disruption of hydrogen bonding and rearrangement of the polymer chains. Smaller lateral crystallite sizes and lower crystallinity contribute to a more amorphous matrix, which can increase the accessible surface area and facilitate dye adsorption. In contrast, larger cellulose crystallites provide mechanical rigidity and structural stability within the hydrogel network, demonstrating a balance between amorphous flexibility and crystalline reinforcement in cellulose-based materials [5,42,43]. In general, the lateral crystallite sizes of chitosan were more dispersed, ranging from 1.99 to 5.1 nm depending on the plane, which indicates a less compact organization and a more irregular structure. This difference can be attributed to the presence of amino groups and to the greater flexibility of the chitosan chains, both of which hinder regular packing and promote the formation of amorphous regions [44]. These results suggest that cellulose acts as the main structural component, showing less variation in crystallite size across its planes and thus providing rigidity and dimensional stability to the system.

The FTIR spectra of the hydrogels (Figure S1, Supplementary Materials) only exhibit the bands characteristic of cellulose and chitosan, with no new peaks attributable to additional covalent bonding between the polymers, supporting the formation of a physically cross-linked network.

### 3.3. Antimicrobial Activity and Biofilm Inhibition of Cellulose–Chitosan Aerogels

The antimicrobial performance of the cellulose–chitosan aerogels was evaluated against a Gram-negative strain (*Escherichia coli*) and a Gram-positive strain (*Staphylococcus aureus*), considering both planktonic cells and biofilm formation after 24 h of incubation.

The antimicrobial activity and biofilm inhibition of all aerogels are shown in Table 4. At 24 h, all aerogel compositions produced a marked inhibitory effect against *S. aureus*, achieving reductions above 99.9% in planktonic cultures relative to the control, which is compatible with a bactericidal response. The strong susceptibility of the Gram-positive strain can be related to the electrostatic attraction between the positively charged amino groups of chitosan and the predominantly negative charges present in the bacterial cell envelope, which promotes membrane disruption and leakage of intracellular components [45]. Biofilm-associated cells of *S. aureus* were also strongly reduced and followed the same order of magnitude as their planktonic counterparts for each aerogel, indicating that these materials interfere not only with free-cell proliferation but also with the establishment of adherent communities on their surfaces. This observation agrees with typical time–kill profiles reported for polymer-based antimicrobial materials, where significant population reductions are often detected only after extended exposure, once sufficient interaction between bacteria and the material surface has occurred [33].

The response of *E. coli* was strongly dependent on the cellulose–chitosan ratio. Aerogels S1, S3 and S5 led to inhibitory rates higher than 99.9% for planktonic cells at 24 h, indicating a bactericidal effect, whereas S2 and S4 reached around 96% inhibition and did not surpass the ≥99.9% threshold. This composition-dependent behavior mirrors the trends previously observed in dye adsorption, where mixed systems exhibited strong interactions with anionic Congo red due to the cationic nature of chitosan, while cellulose-rich samples maximized methylene blue uptake through their hydroxyl groups. In the antimicrobial assay, the most effective compositions corresponded to either chitosan (S1) or cellulose (S5) aerogels, as well as the balanced 50:50 system (S3), where chitosan domains remain accessible at the outer surface supported by a highly porous cellulose backbone, thereby enhancing direct contact with negatively charged bacterial cells.

Intermediate compositions (S2 and S4) showed reduced bactericidal performance against *E. coli* despite high overall inhibition, which may reflect less efficient exposure of chitosan active sites and a different distribution of the cellulose–chitosan complexes at the aerogel surface. As previously discussed for dye adsorption and XRD, chitosan tends to encapsulate cellulose fibers and it becomes more amorphous upon interaction, whereas cellulose retains more ordered crystalline domains that provide structural rigidity. The present antimicrobial results suggest that subtle changes in this supramolecular arrangement affect the accessibility of cationic chitosan segments and thus the intensity of electrostatic interactions with bacterial membranes, in agreement with structure–function correlations reported for other cellulose–chitosan composites [10,32].

For both *E. coli* and *S. aureus*, the magnitude of reduction observed in planktonic cells was reflected in the biofilm-associated populations, indicating that the aerogels exert a comparable inhibitory effect on free and attached cells rather than selectively targeting only one growth mode. This behavior can be related to the highly porous architecture and a high water retention capacity of the aerogels, which facilitate diffusion of nutrients and antimicrobial moieties throughout the matrix while providing a charged interface that perturbs bacterial adhesion and microcolony development. It is also consistent with previous reports on functional cellulose-based aerogels, where high porosity and accessible surface charge have been associated with efficient contact-mediated antibacterial activity [46].

It should be noted that some treatments, particularly S4 and S5, exhibited relatively high standard deviations in the CFU counts at 24 h, both for Gram-positive and Gram-negative strains, suggesting a certain variability in the antimicrobial response that should be accounted for when defining target applications. Nevertheless, when integrated with the density, porosity, dye adsorption and crystallinity data, the antimicrobial performance confirms that cellulose–chitosan aerogels can be regarded as multifunctional porous supports, in which mechanical integrity, charge distribution and fluid retention jointly modulate both adsorption and antibacterial properties rather than dictate a single dominant function. The present assay therefore provides complementary evidence of the potential of these bio-based aerogels for advanced environmental and possibly biomedical uses, in line with recent studies on biopolymer-reinforced cellulose aerogels displaying both catalytic and antimicrobial activities [12,32,47].

## 4. Conclusions

Cellulose pulp and chitosan were successfully dissolved and regenerated in an ionic liquid at different ratios, yielding highly porous hydrogels with porosity values between 94.6 and 98.6%. These materials exhibited a clear selective dye uptake under 24 h exposure, as chitosan-rich and intermediate cellulose–chitosan compositions preferentially adsorbed the anionic dye Congo red over a wide concentration range, while the fully cellulose hydrogel maximized the adsorption of the cationic dye methylene blue at intermediate and high equilibrium concentrations. The pronounced decrease in methylene blue uptake observed for hydrogels containing chitosan is consistent with the interaction between the positively charged amino groups of chitosan and the negatively charged hydroxyl groups of cellulose, which reduces the availability of effective binding sites for the cationic dye.

XRD analysis revealed that the crystallinity of chitosan decreases sharply upon interaction with cellulose, while cellulose preserves and even increases its crystalline domains in mixed systems, thereby providing the main contribution to the structural rigidity of the network. Together, both polysaccharides generate a hybrid architecture in which crystalline cellulose domains act as a mechanical backbone and amorphous chitosan domains introduce interfacial interaction capacity, favoring cohesion between phases and modulating functional properties such as porosity, swelling behavior and dye adsorption. Collectively, the XRD, density, and porosity data indicated that crystalline cellulose domains act as a rigid, highly porous backbone, whereas chitosan becomes more amorphous and forms a continuous phase that wraps the cellulose network. This hybrid architecture rationalizes the observed functional behavior, as cellulose-rich domains supply accessible hydroxyl groups and structural stability that favor methylene blue adsorption, while chitosan-rich domains introduce cationic sites and enhanced interfacial interaction capacity, favoring cohesion between phases, swelling behavior and Congo red uptake.

In addition to their adsorption performance and structural features, the cellulose–chitosan aerogels displayed broad-spectrum antimicrobial activity, with all formulations reaching bactericidal activity (≥99.9% inhibition) against *S. aureus* and selected compositions (S1, S3 and S5) also achieving this threshold against *E. coli*, highlighting their potential as multifunctional bio-based materials for environmental remediation and related applications.

Overall, the variations observed in crystallinity, lateral crystallite size, porosity, dye adsorption behavior and antimicrobial performance reflect the structural order that governs the stability and function of the composite hydrogels. Cellulose–chitosan systems therefore demonstrate a clear complementarity, where amorphous chitosan encapsulates crystalline cellulose and the resulting porous, charged network underpins both adsorption and antimicrobial functions. In view of their high porosity, tunable charge distribution, selective removal of anionic and cationic dyes and strong antimicrobial effect, these aerogels can be considered promising bio-based sorbents for dye-contaminated water streams and as antimicrobial porous supports in applications where simultaneous contaminant removal and inhibition of microbial growth are required.

## Figures and Tables

**Table 1:** Cellulose–chitosan compositions of the hydrogels prepared at different cellulose/chitosan mass ratios: S1 (0/100), S2 (25/75), S3 (50/50), S4 (75/25) and S5 (100/0).

Sample	Cellulose (%)	Chitosan (%)
S1	0	100
S2	25	75
S3	50	50
S4	75	25
S5	100	0

**Table 2:** Density and porosity of cellulose/chitosan hydrogels: S1 (0/100), S2 (25/75), S3 (50/50), S4 (75/25) and S5 (100/0).

Samples	Density (g/cm^3^)	Porosity (%)
S1	0.081 ± 0.02	94.6 ± 1.5
S2	0.065 ± 0.01	97.5 ± 0.2
S3	0.054 ± 0.02	98.5 ± 0.9
S4	0.065 ± 0.02	98.0 ± 0.1
S5	0.065 ± 0.01	95.2 ± 1.5

**Table 3:** Crystallinity indices and lateral crystallite sizes of cellulose (C) and chitosan (Cs) in cellulose–chitosan aerogels with different cellulose/chitosan mass ratios: S1 (0/100), S2 (25/75), S3 (50/50), S4 (75/25) and S5 (100/0).

Sample	CrI CII (%)	C L _(110)_	C L _(100)_	C L _(020)_	CrI Cs (%)	Cs L _(020)_	Cs L _(200)_	Cs L _(220)_
S1	-	-	-	-	37 ± 3	2.2 ± 0.2	1.99 ± 0.07	2.7 ± 0.1
S2	18.5 ± 0.3	3.1454 ± 0.0006	2.7 ± 0.3	4.2 ± 0.2	12 ± 4	3.3 ± 0.2	3.1 ± 0.5	3 ± 1
S3	18.1 ± 0.2	3.28 ± 0.06	3.1 ± 0.4	3.9 ± 0.7	10 ± 2	4 ± 1	3 ± 0.4	3.1 ± 0.6
S4	24.8 ± 0.3	4.8 ± 0.8	2.396 ± 0.001	3.1 ± 0.1	7.8 ± 0.1	5.1 ± 0.3	2.393 ± 0.001	3.3 ± 0.4
S5	29.3 ± 0.9	3.04 ± 0.35	3 ± 0.2	3.3 ± 0.1	-	-	-	-

**Table 4:** Antimicrobial activity and biofilm inhibition of cellulose–chitosan aerogels with different cellulose/chitosan mass ratios, S1 (0/100), S2 (25/75), S3 (50/50), S4 (75/25) and S5 (100/0), against *Escherichia coli* and *Staphylococcus aureus* after 24 h of incubation.

Bacterial Strain	Sample	Viable Cell Count (CFU/mL)		Inhibitory Rate (%)	
		Planktonic	Biofilm	Planktonic	Biofilm
		24 h			
*Escherichia* *coli*	Control	(2.1 ± 0.2) × 10^9^	(2.73 ± 0.09) × 10^8^	0	0
	S1	(2 ± 3) × 10^4^	(2.6 ± 0.5) × 10^4^	>99.99	99.99
	S2	(8 ± 8) × 10^7^	(1 ± 2) × 10^7^	96.43	94.96
	S3	(2 ± 1) × 10^3^	(8 ± 7) × 10^3^	>99.99	>99.99
	S4	(8 ± 8) × 10^7^	(2 ± 2) × 10^7^	96.43	93.44
	S5	(3 ± 4) × 10^3^	(1 ± 0.2) × 10^3^	>99.99	>99.99
*Staphylococcus aureus*	Control	(1.1 ± 0.8) × 10^10^	(2.5 ± 0.2) × 10^8^	0	0
	S1	<2.5 × 10^2^	(3 ± 1) × 10^1^	>99.99	>99.99
	S2	(8 ± 8) × 10^6^	(10 ± 1) × 10^6^	99.93	96.17
	S3	(2 ± 2) × 10^5^	(6 ± 6) × 10^6^	>99.99	97.79
	S4	(9 ± 10) × 10^4^	(7 ± 6) × 10^4^	>99.99	99.97
	S5	(1 ± 1) × 10^3^	(3 ± 2) × 10^4^	>99.99	99.99

## Data Availability

The data that support the findings of this study are available within the article and its Supplementary Materials. Additional raw data are available from the corresponding author upon reasonable request.

## Supplementary Materials

The following supporting information can be downloaded at: https://www.mdpi.com/article/10.3390/polym18131649/s1. Figure S1. FTIR spectra of cellulose-chitosan hydrogels S1–S5.
