# Supplementary material for: Selective Dye Adsorption and Antimicrobial Performance of Cellulose–Chitosan Hydrogels and Aerogels: Role of Supramolecular Organization
Source: Polymers (Basel). 2026 Jul 2;18(13):1649. doi: 10.3390/polym18131649 (PMC13364130; doi:10.3390/polym18131649)
Supplement: Supplementary file 1 [file polymers-18-01649-s001.zip › polymers-4391257-supplementary.pdf]

Supplementary information

Selective Dye Adsorption and Antimicrobial Performance of Cellulose-Chitosan Hydrogels and Aerogels: Role of Supramolecular Organization

Methodology

Fourier transform infrared (FTIR-ATR). FTIR analysis was used to determine the functional groups present in the cellulose-chitosan aerogels. The samples were analyzed using an IRAffinity-1S Shimadzu FTIR spectrophotometer coupled with a Shimadzu attenuated total reflectance (ATR) accessory (Shimadzu Co., Japan). Spectra were recorded in the range 400-4000 cm^{-1} with a resolution of 4 cm^{-1} and 64 scans per sample. The raw spectra were baseline-corrected, and the baseline was subtracted over the selected points; a Savitzky-Golay smoothing filter of 50 points was then applied using OriginPro 9 (OriginLab Corp., USA) to improve the signal-to-noise ratio and facilitate comparison between compositions.

Results

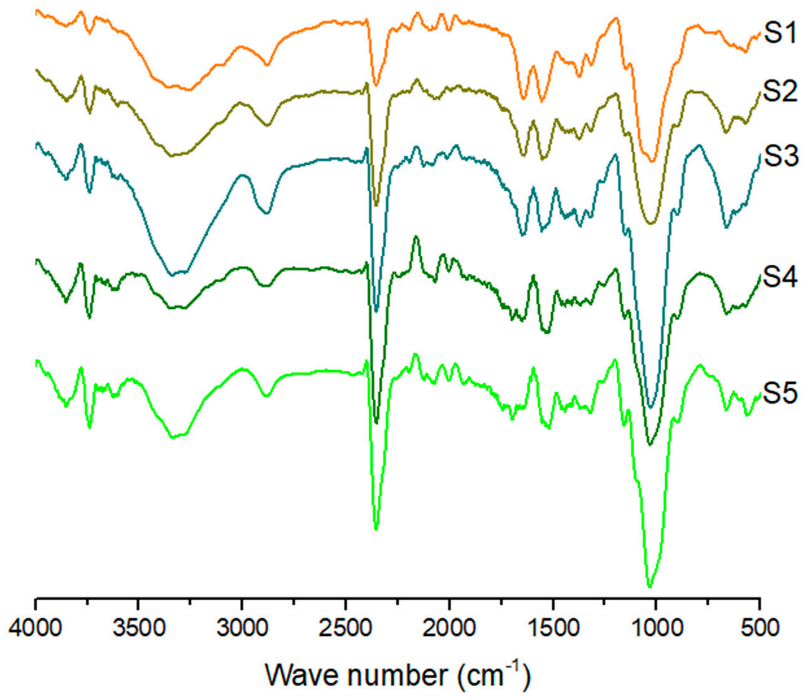

Figure S1. FTIR spectra of hydrogels with different cellulose/chitosan mass ratios: S1 (0/100), S2 (25/75), S3 (50/50), S4 (75/25) and S5 (100/0). respectively.

The FTIR spectra of S1-S5 (Figure S1) show the characteristic bands of cellulose and chitosan, with no new peaks attributable to covalent bond formation between the two polymers. The main absorption bands include a broad O-H/N-H stretching vibration centered at approximately 3300 cm^{-1}, C-H stretching at 2900 cm^{-1}, amide I (C=O stretching) at 1640 cm^{-1}, amide II (N-H bending) at 1550 cm^{-1}, and C-O-C stretching in the 1000-1100 cm^{-1} region. Notably, slight shifts in the O-H/N-H stretching band (3300-3350 cm^{-1}) and changes in intensity of the amide I and amide II bands were observed as a function of composition, consistent with hydrogen-bonding interactions between cellulose and chitosan chains. This behavior supports the interpretation that the hydrogels networks are stabilized by physical interactions (hydrogen bonding and electrostatic interactions) rather than covalent cross-linking.
